# Early markers of heart damage in systemic sclerosis: role of cardiac magnetic resonance with late gadolinium enhancement

**DOI:** 10.1186/1532-429X-18-S1-P271

**Published:** 2016-01-27

**Authors:** Chiara Valentina Lario, Federica Ferraris, Annalisa Balbo Mussetto, Annalisa Macera, Raffaele Pellerito, Stefano Cirillo

**Affiliations:** grid.414700.60000000404845983Ospedale Mauriziano, Torino, Italy

## Background

Cardiovascular complications are one of the main causes of death in patients affected by systemic sclerosis (SSc). Heart involvement is characterized by myocardial fibrosis, arrhythmias, conduction system defects and pericardial inflammation. Also patients with subclinical cardiac involvement have a poor prognosis: thus early markers of heart damage are necessary to identify patients at risk. Left ventricle (LV) diastolic dysfunction evaluated with echocardiography has recently been considered as an early marker of cardiac involvement and myocardial fibrosis in SSc patients. As cardiac magnetic resonance (CMR) with late gadolinium enhancement (LGE) allows an in vivo, non invasive, tissue characterization and detection of myocardial fibrosis, we evaluate the presence and extension of LGE in a population of SSc, the relationship with echocardiography diastolic dysfunction and clinical parameters to individuate a possible role of CMR in early detection of heart disease in SSc patients.

## Methods

we studied 43 consecutive SSc patients and excluded those with significant cardiac involvement, arterial hypertension, diabetes mellitus, renal failure, pulmonary hypertension and contraindication to perform a CMR. We divided the study sample into those with and without LGE. The quantification of LGE was made with a semi quantitative method: enhanced myocardium was defined with a cut-off of 6SD above the mean of ROI (placed in normal remote myocardium).

## Results

42% of patients had LGE (65% of patients had diastolic dysfunction). The average percentage of LGE extension was 6% (5 g of myocardial mass). The logistic regression showed no correlation between amount of LGE and the presence of diastolic dysfunction and at the one-way analysis of variance, no correlation was demonstrated also between the presence of LGE and diastolic dysfunction. LGE was correlated to an increased LV mass and right ventricle dilatation. At follow-up (average time of 16 months), LGE was correlated to progressive increase in pulmonary arterial pressure evaluated with echocardiography.

## Conclusions

In our study, CMR in a sample of SSc patients showed a high incidence of LGE, with limited extension (6% of myocardium on average). We did not find a correlation between the presence and extension of LGE at CMR and the diastolic dysfunction at echocardiography. So the preliminary data of our study did not allow LGE to be considered a marker of diastolic dysfunction in SSc patients.

Important we demonstrate the relationship between LGE and a progressive increase in pulmonary arterial value at follow-up: this is relevant due to the clinical implication of pulmonary artery hypertension in SSc patients and suggests (but not prove) that LGE allow to individuate a group of SSc patients at risk of develop pulmonary artery hypertension over time.

In this context, if these data will be confirmed by a study on a larger sample of patients and with a longer follow-up, LGE could be considered a possible early marker of heart damage.

